# Editorial: Role of assistive technologies to offer diagnosis, intervention and rehabilitation for individuals with neurological or neurodevelopmental disorder

**DOI:** 10.3389/fpsyt.2023.1159267

**Published:** 2023-04-11

**Authors:** Kazunori Miyata

**Affiliations:** Graduate School of Advanced Science and Technology, Japan Advanced Institute of Science and Technology, Nomi, Japan

**Keywords:** Developmental Language Disorder, Music Interventions, dementia, wearable device, delirium, participatory design

Recent developments in computer technology have led to dramatic advances in assistive technologies for people with neurological and neurodevelopmental disorders. In particular, advances in sensing and HCI technologies have been shown to help provide real-time intervention and rehabilitation support for people with disabilities and diseases. Since the COVID-19 pandemic, most in-person intervention and rehabilitation has been affected, and the use of ICT to support and monitor patients and people with disabilities will become increasingly important.

Adolescents with developmental language disorder (DLD) have more problems with social-emotional functioning than typically developing adolescents, including poor peer relationships, anxiety, victimization and social isolation. To develop effective support for adolescents with DLD, it is necessary to better understand the factors underlying their social-emotional problems. The review, “*Improving social emotional functioning in adolescents with developmental language disorders: A mini review and recommendations*,” states that interventions to improve social-emotional functioning in adolescents with DLD should target the language, social communication skills and cognitive components that underlie social-emotional functioning (Arts et al.). In addition, interventions should provide opportunities for explicit practice of these skills through interaction with others and promote internalization. This could be achieved through the use of media technologies such as VR.

One of the challenges in the clinical management of older people with dementia is monitoring their agitation. Wrist accelerometers may be a useful tool for this monitoring. The review, “*Wrist accelerometry for monitoring dementia agitation behaviour in clinical settings: A scoping review*,” shows that the frequency or entropy (aggregate energy) of activity levels measured by wrist accelerometers has been shown to be associated with agitated behavior in clinical settings, but that their performance in identifying the occurrence of agitated episodes is unsatisfactory (Cheung et al.). They recommended that the protocol for the assessment of agitated behavior should be standardized by unifying the parameters to be measured, cut-offs, measurement periods and sampling windows for wrist accelerometers. They also suggested that multimodal data could improve the accuracy of agitation evaluation.

Dementia is the only disease in the top 10 causes of death worldwide that is difficult to prevent or treat, and a range of innovations in disease management, caregiver support and services are needed to reduce the burden on those involved. Even in dementia, musical memories of familiar songs and genres are often well-preserved and can engage attention and improve mood. Personal connections to favorite music can also support a positive sense of identity in later stages of the disease. Music interventions for people with dementia can promote health and interaction with caregivers, but are often limited to institutional settings. The paper, “*The acceptability, adoption, and feasibility of a music application developed using participatory design for home-dwelling persons with dementia and their caregivers. The ‘Alight' app in the* *LIVE@Home.Path* *trial*,” describes a participatory design process for a prototype music application for patients in a geriatric psychiatric hospital. Mobile health (mHealth), as defined by the WHO, can extend the reach of music interventions, but there are barriers for people with dementia, including digital literacy and design issues (Berge et al.). In contrast, this paper developed the application through a participatory design process involving patients and carers. Initial prototyping and testing suggested its use as a “digital music memory book” for people with dementia and suggested that the music intervention could be delivered to people with dementia and their carers at home *via* an mHealth application with high acceptability, adoption and feasibility. After the developed application was validated in LIVE@Home.Path trial, they confirmed that the feasibility and adoption of the application was high, and that accepting dyads did not differ on demographic and clinical variables from those who were not reached. This suggests a high potential for use in dementia care.

Intensive care units (ICUs) are places where a variety of medical staff and patients with different conditions come together. A delirium prediction model that can be easily applied would be useful for the management of delirium in ICUs.

The paper, “*Development and validation of simplified delirium prediction model in intensive care unit*,” attempts to develop a delirium prediction model that can be easily used by different medical staff (Kim et al.). Specifically, we used the occurrence of delirium within 30 days of ICU admission as the output variable and 12 simple predictors such as restraint, admission route and drainage tube placement. Two prediction models were developed using logistic regression (LR) and random forest (RF). After validation, the LR method was selected as the final model because of its high discriminatory power based on the area under the receiver operating characteristic curve (AUROC). A key feature of this study is that no special tests were performed and only simple factors were used as predictors. This simplified model makes it easier for clinicians to carry out screening and allows for more effective and proactive interventions to prevent delirium.

## Author contributions

The author confirms being the sole contributor of this work and has approved it for publication.

